# High-sensitive Troponin T increase after exercise in patients with pulmonary arterial hypertension

**DOI:** 10.1186/1471-2466-13-28

**Published:** 2013-04-29

**Authors:** Mirko Völkers, David Rohde, Thomas Zelniker, Celine S Weiss, Evangelos Giannitsis, Hugo A Katus, F Joachim Meyer

**Affiliations:** 1 Department of Cardiology, Angiology and Respiratory Medicine, University Medical Center, Heidelberg, D-69120, Germany; 2 Department of Respiratory Medicine, Gastroenterology and Internistic Intensive Care, Munich Municipal Hospital at Harlaching, Munich, Germany

**Keywords:** High-sensitive Troponin T, Pulmonary arterial hypertension, Cardiopulmonary exercise testing

## Abstract

**Background:**

The current study aimed to investigate the release of myocardial high-sensitive Troponin T (hsTnT) in patients with pulmonary arterial hypertension (PAH) in response to maximal physical exercise.

**Methods:**

In 24 patients with PAH, symptom-limited cardiopulmonary exercise testing was performed. hsTnT was measured by the novel hsTnT assay with a lower limit of detection of 2 ng/L and a total imprecision of less than 10% at the 99^th^ percentile value. hsTnT was related to NT-proBNP, WHO functional class and right ventricular (RV) function. Serial measurement was performed before and 30 min, 180 min, and 300 min after exercise. Healthy volunteers served as a control group.

**Results:**

In 21 PAH patients, hsTnT levels were detectable before exercise with a close correlation between hsTnT and NT-proBNP. hsTnT was detectable in all PAH patients after exercise and significantly increased from 7.5 ng/L at baseline to 14.62 ng/L after 300 min, whereas levels of NT-proBNP remained constant with time.

**Conclusions:**

Using the novel hsTnT assay, the current study provides first evidence that hsTnT levels increase in PAH patients after maximal physical exercise, while levels of other biomarkers remain constant after exercise testing. This might provide new insights into pathophysiology and individual risk assessment in patients with PAH.

## Background

Pulmonary arterial hypertension (PAH) is a progressive disease leading to reduced functional status with a poor prognosis [1]. A high pulmonary vascular resistance and right ventricular dysfunction impair stroke volume of the right ventricle leading to impaired functional capacity. Compared to the National Institutes of Health-supported 1980s registry [2], substantial progress has been achieved in the treatment of PAH due to improved pharmacotherapy with drugs targeting different molecular targets since.

Exercise training in PAH patients used to be considered as potentially hazardous. However, the first clinical trial on exercise training in patients with PAH reported improved exercise capacity and quality of life [3]. Several follow-up studies confirmed that exercise training improved endurance and peripheral muscle function in patients with PAH and supported the role of exercise training as an adjunct therapeutic regime [3,4]. Moreover, recent studies with larger patient populations approved safety and efficacy of closely monitored exercise training in various forms of pulmonary hypertension, though Grünig et al. characterized it as potentially harmful due to the risk of adverse events [5]. However, negative results of training have been reported in an experimental model of PAH, where exercise training was beneficial in stable PAH, but detrimental in progressive PAH [6]. Today, recommendations regarding type, frequency or intensity of exercise training are not enclosed in the current guidelines of PAH treatment.

Cardiac Troponin T (cTnT) is the preferred biomarker for detection of myocardial cell injury [7]. Well-known reasons for increased cTnT levels include irreversible myocardial necrosis in patients with acute coronary syndrome as well as direct or indirect myocardial cell damage due to non-ischemic injury such as toxins or infections. High-sensitive Troponin T (hsTnT) assays have been recently introduced and are characterized by increased analytical sensitivity and the ability to measure concentrations at the 99^th^ percentile on a reference population with an imprecision of less than 10% [8]. Moreover, the concentrations of cardiac markers such as cTnT are known to increase after prolonged exercise to levels seen after minor myocardial infarction [9]. Two mechanisms of elevated cTnT values after prolonged exercise have been postulated and include either an increase in myocardial injury due to the true breakdown of myocytes or the cytosolic release of the biomarker [10,11].

Recently, we showed that elevated concentrations of hsTnT predict advanced WHO functional class and death in patients with PAH [12]. Elevated levels of hsTnT showed a close relation with systolic RV dysfunction. We designed the study to determine if patients with PAH in comparision with healthy volunteers develop significant hsTnT release after symptom-limited cardiopulmonary exercise testing.

## Methods

This study enrolled 24 patients with PAH. All patients were treated according to current guidelines for PAH treatment. At time of the exercise testing patient were clinically stable since at least 4 weeks and none showed clinical signs of cardiopulmonary decompensation. Patients with severe renal failure (creatinine clearance <60 mL/min/1.73 m^2^) were excluded.

Twelve healthy volunteers served as a control cohort. Control subjects had no suspicion of any pathological finding in echocardiography at rest, body-plethysmography and symptom-limited cardiopulmonary exercise testing. Addtionally, all relevant laboratory values (including hsTnT, NT-proBNP and creatinin) were within standard values.

### Study population

This study was conducted in a university tertiary referral center for patients with PAH (Department of Cardiology and Respiratory Medicine, Medical Center, University Hospital, Heidelberg, Germany) and included patients with idiopathic PAH (IPAH) and chronic-thrombembolic pulmonary hypertension (CTEPH) [13]. The diagnosis of IPAH was made after right heart catheterization, and ventilation-perfusion scan, spiral computer tomography, three-dimensional angiography magnetic resonance tomography, or pulmonary angiography to rule out pulmonary embolic etiology, and after exclusion of underlying autoimmune disease, collagen vascular disease, hepatic or HIV infection, and nocturnal deoxygenation. The local ethics committee approved the study and all patients gave written informed consent prior to inclusion. The study was designed and performed in accordance with the recommendations found in the Helsinki Declaration.

### Right heart catherterization

Right heart catheter was performed with a Swan-Ganz catheter from either the right internal jugular or right femoral vein as reported previously [14]. The zero reference pressure has been estimated in a plane 5 cm dorsally to the sternal angle. PAPm and PCWPm were measured in the supine position at rest. Cardiac output (CO) measurements were obtained using the thermodilution method. CO was calculated as the mean value from 3 measurements with <10% variability of at least 5 measurements. The measurements were made using the mean at end-expiration, and were analyzed by two independent investigators from the raw data.

### Cardiopulmonary exercise testing

Cardiopulmonary exercise testing (CPET) was performed using a progressively increasing work rate to maximum tolerance on an electromagnetically braked semi-reclining cycle ergometer (ergoselect 1000, ergoline GmbH, Bitz, Germany) with an integrated complete cardiorespiratory diagnosis system (MasterSreen™ CPX, CareFusion, Hoechberg, Germany). Heart rate, ECG, ventilation, carbon dioxide output, oxygen uptake, work rate, end-tidal carbon dioxide and end-tidal oxygen pressure, as well as other gas exchange variables were measured continously as described before [3]. From these data, the peak oxygen uptake, peak heart rate, peak oxygen pulse, respiratory exchange ratio (which equals carbon dioxide output/oxygen uptake), anaerobic threshold, ventilatory equivalents for oxygen and carbon dioxide were calculated. The anaerobic threshold was detected with the V-slope method [15].

### Serum biomarkers

Blood samples were drawn from a peripheral vein. Samples were stored immediately at -80°C. Samples were later analyzed in batches. hsTnT was measured using the new hsTnT quantitative electrochemiluminescence immunoassay (Roche Diagnostics, Mannheim, Germany) as described previously [12]. The assay is specific for Troponin T without relevant interferences and has an analytic range of 3–10000 ng/L (limit of the blank/LOB 3 ng/L, limit of quantification/LOQ 14 ng/L). A concentration of 14 ng/L has been identified as the 99th percentile of a healthy reference population with a CV of <10%. Routine laboratory parameter measurements including NT-proBNP and creatinine were performed at the core laboratory of the University Hospital of Heidelberg.

### Six-minute walk test

The results of the six-minute walk test (6-MWT) were counted from the laps achieved on a 60-m course in a straight hospital hallway. The test equipment and the interaction with the patient were provided as recommended [14].

### Data analysis

Statistical analysis was performed by a professional statistician using standard software (SAS 9.1 WIN). Results are expressed as mean ± standard deviation (SD). Paired and unpaired Student's *t*-test and Pearson's correlation coefficient were analyzed as appropriate. P-values < 0.05 were considered statistically significant.

## Results

The baseline characteristics of the patients are presented in Table 1. We included 24 patients with PAH. PH was idiopathic in 15 patients and thromboembolic in 9 patients. Among these 24 patients, 15 patients (62%) were in WHO functional class II and 9 patients (38%) in WHO functional class III. The mean pulmonary arterial pressure was 48.72 ± 18.69 mmHg. The peak oxygen uptake after maximal exercise on standardized spiroergometry was 13.32 ± 3.3 VO_2_ mL/kg/min and severely impaired compared to the control cohort. To compare the effect of symptom-limited physical exercise on hsTnT we included 12 healthy volunteers with no known cardiopulmonary diseases. The control cohort was younger and achieved a significantly higher maximal workload ( 244.58 ± 60.56 vs 74.61 ± 21.07 Watt) and Peak VO_2_ (38.73 ± 11.34 vs 13.32 ± 3.3 mL/kg/min) compared with the PAH patients.

**Table 1:** Patients’ and control cohort characteristics

**n = 24**	**Mean ± SD**
Age (years)	57.27 ± 16.33
Male (%)	65
IPAH (%)	60
CTEPH (%)	40
BMI (kg/cm^2^)	27,17 ± 6.45
SvO2 (%)	62.86 ± 7.92
PAmean (mmHg)	48.72 ± 18.69
PVR (dyn*s*cm-^5^)	638.18 ± 381.06
Mean RAP (mmHg)	10.82 ± 4.63
Workload max (Watt)	74.61 ± 21.07
Peak VO2 (ml/kg/min)	13.32 ± 3.3
SysRRmax (mmHg)	153.56 ± 32.42
NT-proBNP (ng/L)	363.39 ± 461.68
6-MWT (m)	428.52 ± 118.96
Ejection fraction (%)	55.13 ± 4.34
Diastolic LV-dysfunction (%)	41.67
History of CAD (%)	12.5
Creatinin (mg/dL)	1.12 ± 0.83
**Control cohort characteristics**	
**n = 12**	**mean ± SD**
Age (years)	36.8 ± 10.7
Male (%)	58
Workload max (Watt)	244.58 ± 60.56
Peak VO2 (ml/kg/min)	38.73 ± 11.34
NT-proBNP (ng/L)	31.65 ± 42.49

**Figure 1:**
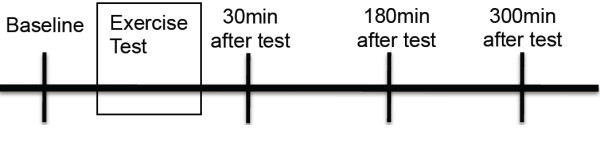
**Schematic flow chart of the study design. **Blood was taken before the exercise and 30 min, 180 min and 300 min after exercise testing.

**Figure 2:**
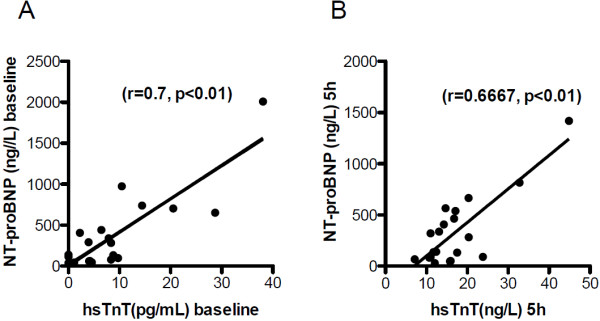
HsTnT correlates with NT-proBNP concentrations in patients with PAH at baseline conditions (A) and 5 hrs after exercise testing (B).

To address the question if symptom-limited exercise increased hsTnT levels in patients with PAH, we took blood samples at 30 min, 180 min and 300 min after CPET. Figure 1 illustrates the study design. Twenty out of 24 patients had detectable hsTnT at baseline before exercise, compared to only 3 out of 12 healthy volunteers. Whereas hsTnT levels were <14 ng/L (limit of quantification) in these control subjects (3.34 ng/L, 3.96 ng/L and 5.97 ng/L), hsTnT baseline values were >14 ng/L and thus pathologically elevated in 4 patients. Elevated hsTnT levels are associated with death and decreased right ventricle function in patients with PAH [14]. We found a close relationship between hsTnT levels and NT-proBNP at baseline (r = 0.7, p < 0.01) as well as 5 hours after maximal exercise (r = 0.667, p < 0.01) (Figure 2A and 2B).

**Figure 3:**
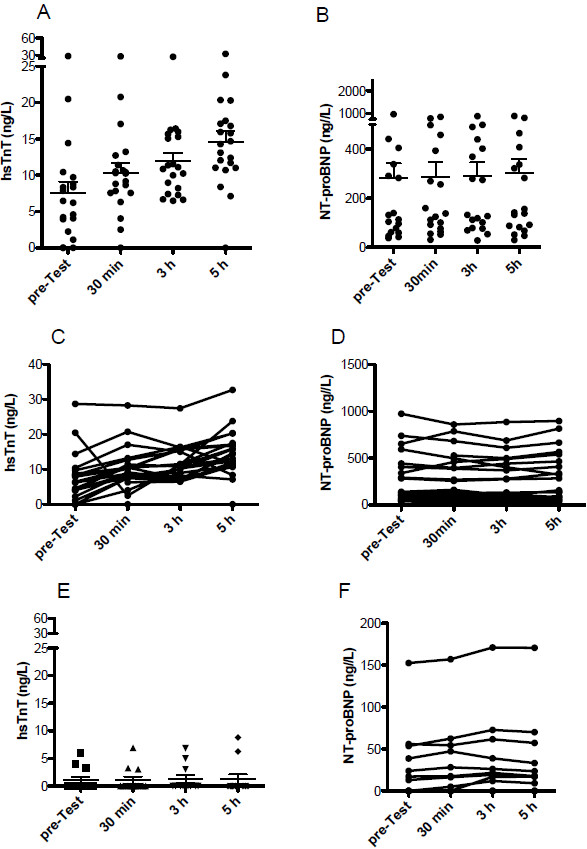
**Plasma levels of hsTnT increased time-dependently after exercise (Scatter Blot A and individual patients C), whereas levels of NT-proBNP remained constant (Scatter Blot B and individual patients D) in patients with PAH. **HsTnT (**E**) and NT-proBNP (**F**) remained unchanged in the healthy control cohort after exercise.

Interestingly, concentration of hsTnT increased in PAH patients after exercise with time and reached significance 5 hours post CPET (Figure 3A and 3C). Serum concentrations of NT-proBNP remained constant after exercise (Figure 3B and 3D). Conversely, no detectable increase in hsTnT serum concentrations was observed in the control cohort. As well, the control cohort had no change in serum concentration of NT-proBNP (Figure 3E and 3F).

**Figure 4:**
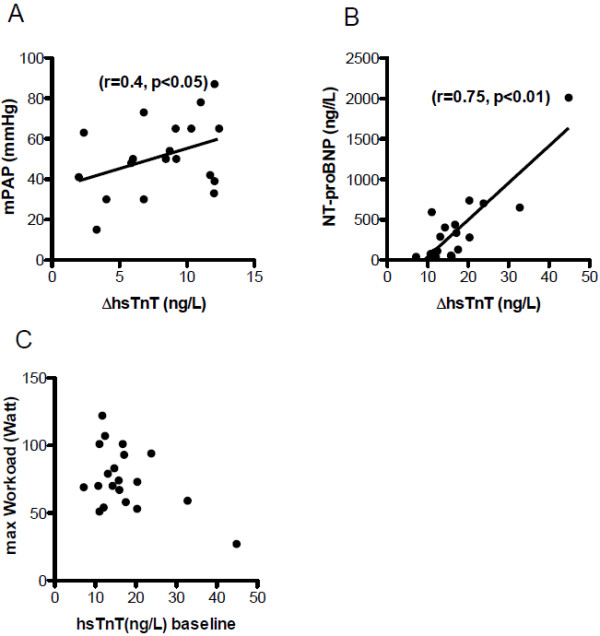
**Correlation between ∆ hsTnT and clinical parameters. **Correlation between ∆ hsTnT and mPAP (**A**) and NT-proBNP (**B**) showed positive correlation whereas ∆ hsTnT and maximal Workload (**C**) showed no correlation.

Next, we correlated the individual increase of hsTnT serum concentrations (∆hsTnT) in each individual PAH patient with the mean pulmonary arterial pressure (Figure 4A) and with serum levels of NT-proBNP (Figure 4B). Interestingly, the severity of the PAH (measured by mean mPAP) correlated with the ∆hsTnT and with the NT-proBNP plasma levels, whereas ∆hsTnT failed to correlated with the maximal workload (Figure 4B) or peak oxygen uptake (data not shown).

## Discussion

To our best knowledge, this is the first study to investigate hsTnT release in PAH patients after cardiopulmonary exercise testing. The major finding of the present study is that symptom-limited exercise using standardized cardiopulmonary exercise testing in PAH patients leads to significantly increased serum level of hsTnT. We demonstrated a time dependent increase of serum levels of hsTnT, which was not detectable in a healthy control cohort.

The role of physical exercise in current clinical management of patients with pulmonary hypertension is a continously discussed topic. Traditionally, these patients were advised to limit their physical activity. Exercise was thought to increase the workload for the already compromised right ventricle. Recently, a prospective study by Merelez et al. [3] showed that exercise training remarkably improved exercice capacity and quality of life in clinically stable patients. In accordance, another study demonstrated exercise training improved endurance and muscle function [1]. However, it remains uncertain if over-ambicious exercise training with repeated symptom-limited physical activity can contribute to myocardial damage in PAH patients. Interestingly, a recent study showed that exercise training was detrimental in mice with progressive PAH, resulting in adverse effects on hemodynamics and accelerated the progression of right heart failure [4].

It is well known that RV systolic dysfunction transiently occurs after prolonged physical exercise i.e. marathon running in endurance athletes. It has been shown in several studies that cardiac biomarkers are elevated after strenuous exercise such as marathon [9,16-18]. The transient RV systolic dysfunction was linked to exercise induced pulmonary hypertension [19]. Therefore it is tempting to speculate that in PAH patients the elevated pulmonary arterial pressure is causal for the elevated hsTnT after exercise. This is also supported by the fact that healthy volunteers showed no increase in hsTnT after symptom-limited brief exercise. However, it cannot be excluded from the data raised in this study, whether comorbid left ventricular systolic or diastolic dysfunction or coronary artery disease may have contributed to hsTnT elevation in some PAH patients.

Controversies exist regarding the mode of Troponin T release, i.e. membrane leakage vs. cardiomyocytes necrosis. The first hypothesis is supported by the finding of short and quantitatively small Troponin release in patients with significant pulmonary embolism [11,20]. Other findings support the hypothesis that Troponin release is caused by cardiomyocyte necrosis. Immunhistochemical studies have demonstrated that cardiac Troponin is only released after irreversible cardiomyocyte damage [10]. However, low serum concentrations of Troponin T are detectable in healthy subjects with the new hsTnT quantitative electrochemiluminescence immunoassay [21], as observed in our control cohort. Although values <14 ng/L are not considered as pathological, the mechanism of Troponin T release in those subjects is currently not known. In summary the underlying pathological mechanisms for the increase in hsTnT in our patients remains to be elucidated by further studies.

It should be noted that the majority of our patients belonged to the WHO FC II and was clinically stable at the time of the examination in order to tolerate the exercise test. We were able to detect hsTnT in 83% of our patients with PAH at rest. All patients showed increased concentrations of hsTnT after exercise and the relative increase of hsTnT correlated positively with both mPAP and serum levels of NT-proBNP. Therefore we speculate that the release of hsTnT after exercise is dependent on RV function and increased right ventricular wall stress in patients with more severe PAH. This finding could be of clinical relevance as maximal exercise in patients with severe PAH and progressive right ventricular failure could cause a clinically significant increase in hsTnT.

However, there are some limitations in regard to interpreting the study results. 21 of the 24 PAH patients analysed had hsTnT levels that were above the lower level of detection (LOB 3 pg/mL) but below the level of quantification (LOQ 14 pg/mL) before exercise. Due to measurement uncertainty, hsTnT levels at this level remain to be of clinically questionable relevance. Nevertheless, 12 patients with initial hsTnT > 3 pg/mL < 14 pg/mL converted to serum concentrations > 14 pg/mL after CPET. Moreover, there was a uniform tendency towards elevated hsTnT values after exercise in all PAH patients which occurred irrespective of the initial hsTnT level.

Yet, some major issues remain to be elucidated. Due to the small sample size and the stable clinical conditions of the enrolled PAH patients, we could not address the question whether increased Troponin release after exercise predicts fatal events or clinical worsening. Future follow up studies will address these questions. Moreover, hsTnT was serially measured after symptom-limited and very short exercise. This does not reflect controlled exercise and respiratory training used in previous clinical studies. If controlled exercise-training can cause relevant hsTnT release in patients with progressive RV dysfunction remains to be investigated. However, many patients with severe PAH want to exercise more than they are allowed to and we expect that further research is required to optimize training programs for PAH patients at early and late stages.

## Conclusions

The current study demonstrates for the first time that serum levels of hsTnT increased after symptom-limited exercise testing in patients with PAH. Our study supports the role of increased right ventricular wall stress in the release of cardiac Troponin in PAH. Exercise testing with assessment of hsTnT might provide new insights into pathophysiology and individual risk assessment in patients with PAH.

## Competing interests

The authors declare that they have no competing interests.

## Authors’ contributions

MV carried out the exercise test, designed the study and drafted the manuscript. DR carried out the exercise test and designed the study. TZ carried out the exercise test. CW carried out echocardiograms. EG participated in the design of the study and performed the statistical analysis. HAK and FJM conceived of the study, and participated in its design and coordination and helped to draft the manuscript. All authors read and approved the final manuscript.

## Pre-publication history

The pre-publication history for this paper can be accessed here:

http://www.biomedcentral.com/1471-2466/13/28/prepub
